# *Ogataea* Yeast Platform for Recombinant Porcine Interferon-Alpha 1 Production: Toward Alternative Biotherapeutics for Swine Health Management

**DOI:** 10.3390/microorganisms14102150

**Published:** 2026-09-23

**Authors:** Benjamas Liwnaree, Thorntun Deangphare, Chinnawat Assana, Kitisak Sansatchanon, Jedsadakorn Ninrat, Jaraspim Narkpuk, Nanchaya Wanasen, Aekkachai Puseenam, Niran Roongsawang, Kanokarn Kocharin, Peera Jaru-Ampornpan

**Affiliations:** 1 Veterinary Health Innovation and Management Research Group, National Center for Genetic Engineering and Biotechnology (BIOTEC), National Science and Technology Development Agency (NSTDA), Pathum Thani 12120, Thailand; benjamas.chu@biotec.or.th (B.L.); thorntun.d_s25@vistec.ac.th (T.D.); chinnawat.ass@ncr.nstda.or.th (C.A.); jaraspim.nar@biotec.or.th (J.N.); nanchaya.wan@biotec.or.th (N.W.); 2 Biorefinery and Bioproduct Technology Research Group, National Center for Genetic Engineering and Biotechnology (BIOTEC), National Science and Technology Development Agency (NSTDA), Pathum Thani 12120, Thailand; kitisak.san@biotec.or.th (K.S.); jedsadakorn.nin@ncr.nstda.or.th (J.N.); aekkachai.pus@biotec.or.th (A.P.); niran.roo@biotec.or.th (N.R.); kanokarn.koc@biotec.or.th (K.K.)

**Keywords:** *Ogataea polymorpha*, yeast expression system, recombinant protein expression, porcine interferon, veterinary biotherapeutics, antivirals

## Abstract

Infectious diseases in swine demand affordable, broad-spectrum antiviral strategies, such as recombinant interferons (IFNs). However, commercial yeast expression platforms often present financial barriers through licensing royalties. To provide a cost-effective regional alternative, we evaluated the domestic, thermotolerant yeast *Ogataea polymorpha* OP39 for producing recombinant porcine IFNs. Recombinant OP39 strains carrying expression cassettes for types I (poIFN-α1), II (poIFN-γ), and III (poIFN-λ3) IFNs were constructed and evaluated for their recombinant protein production performance. Following initial screening, the highest-yielding candidate, poIFN-α1, was selected for bioprocess optimization in shake flasks and a 5 L fermenter. Results showed that OP39 successfully secreted all three IFN types with a relatively low level of hyperglycosylation. Batch fermentation scale-up of r-poIFN-α1 achieved a final protein concentration of 0.45 ± 0.10 mg/mL. Crucially, the OP39-produced r-poIFN-α1 exhibited antiviral efficacy in vitro against economically important porcine viruses including African Swine Fever Virus, Porcine Epidemic Diarrhea Virus and Porcine Reproductive and Respiratory Syndrome Virus. Based on the proof-of-concept results in this study, the OP39 platform can be further developed into a viable, full freedom-to-operate system for local or regional biomanufacturing of biologically active veterinary therapeutics to support livestock health management.

## 1. Introduction

At present, infectious diseases cause significant economic losses to pig farmers, particularly those operating medium- and small-sized farms. With currently available vaccines and drugs, emerging or re-emerging pathogens can still pose imminent threats. The economic losses attributed to African Swine Fever outbreaks in Asia during 2019–2022 highlighted the critical needs for alternative or additional farm management strategies. The development of cost-effective, broad-spectrum antiviral agents or immunostimulants for livestock could provide a valuable alternative approach to complement farm vaccination programs and biosecurity measures.

Interferons (IFNs) play crucial roles in mammalian innate and adaptive immune systems, acting as the first line of defense against viral infections and modulators of immune activity. They are typically classified into three main types based on their receptor specificity and structural characteristics: type I interferons (such as IFN-α and IFN-β), type II interferons (represented by IFN-γ), and type III interferons (including IFN-λ) [1,2,3,4]. Type I IFNs, like IFN-α, are primarily involved in antiviral defense by inducing the expression of numerous interferon-stimulated genes that restrict viral replication [2]. Type II IFN is produced mainly by T lymphocytes and natural killer cells and serves as a key activator of macrophages and enhancer of antigen presentation, thereby bridging innate and adaptive immunity [4]. Type III IFNs, such as IFN-λ, exhibit antiviral properties similar to type I IFNs but act predominantly on epithelial surfaces, providing localized immune protection with fewer systemic effects [3]. For these properties, recombinant interferons have been clinically used in both humans and pets. IFN-α2a and IFN-α2b are used for chronic viral infections such as hepatitis B and C in humans [5]. Recently discovered, IFN-λ have been demonstrated in several clinical trials to provide potential benefits for hepatitis viral infections and COVID-19 treatment or prevention [6]. In veterinary practice, recombinant human, canine and feline interferons have shown efficacy in treating infectious or inflammatory diseases in cats and dogs [7].

As IFN pathways are conserved across animal species, most swine viruses are typically IFN-sensitive. Replication of ASFVs in primary porcine pulmonary alveolar macrophages (PAMs) or Vero cells is markedly reduced when cells are pretreated with recombinant IFN-α and IFN-γ prior to infection [8,9,10]. Similarly, replication of Porcine Reproductive and Respiratory Syndrome Virus (PRRSV) in PAMs is significantly reduced when cells are pretreated with IFN-α [11]. Foot-and-Mouth Disease Virus (FMDV) has been shown to be sensitive to IFN both in vitro and in vivo [12,13]. Recent work reported that an oral application of recombinant poIFNα in piglets can reduce viral load and alleviated symptoms of porcine epidemic diarrhea virus (PEDV) [14]. Beyond antiviral functions, IFNs have been explored as veterinary vaccine adjuvants [15,16], as well as immunomodulators for mitigating stress and enhancing immune competence in piglets during the weaning period [17].

However, practical uses of recombinant IFNs in livestock require a sufficiently low production cost. Microbial fermentation, especially yeasts, can offer cost-effective platforms producing biologically active recombinant interferon. To date, several yeast platforms with different properties have been developed to serve a wider need in heterologous protein expression, including more traditional strains such as *S. cerevisiae*, *Komagataella* sp. (including the widely used commercial strain *P. pastoris*) and *Hansenula polymorpha* (currently *Ogataea polymorpha*) or less traditional strains such as *Schizosaccharomyces pombe*, *Kluyveromyces lactis* and *Yarrowia lipolytica*. Some of these commercial strains’ licensing options can create additional financial barriers for local biotech companies in low-income countries focusing on regional problems. To provide a regional alternative, we have characterized thermotolerant domestic *Ogataea polymorpha* TBRC 4839 (OP39), originally isolated from fermented bagasse in Thailand, as a production host and developed the accompanying expression vectors with full freedom-to-operate [18]. The system has been tested for high-cell density fermentation and heterologous protein expression [19]. However, the suitability of the OP39 system in producing a wide range of high-value proteins, especially with applications in biopharmaceuticals, remains to be demonstrated.

In this work, we explored the OP39 system as a production platform to produce recombinant porcine interferons to assess potentials for regional development into veterinary pharmaceutical products. We designed and test-expressed types I, II and III porcine interferons and demonstrated bioactivities of the resulting recombinant proteins. Based on the yield and its wider potential applications, we focused on type I poIFN (poIFN-α1). After optimization in shake flasks, we developed a bioprocess for a pre-pilot 5 L fermentor. Finally, to confirm the potential of r-poIFN-α1 to be used as biotherapeutics or first-line defense in the swine industry, we demonstrated its biological activity against several porcine viruses.

## 2. Materials and Methods

### 2.1. Generation of Expression Vectors

Amino acid sequences for mature porcine interferon proteins (NCBI accession codes: Q5U8T3, ACZ63405.1 and NP_999113.1; signal peptide coding sequences annotated or predicted by UniProt or SignalP program) were used for DNA codon-optimization and commercial synthesis by GenScript Biotech (Singapore). Sequences are provided in Supplementary Figure S1 and Table S1. The genes were PCR-amplified with EcoRI and SacI restriction sites as overhangs. Subsequently, they were cloned into the in-house expression vector pOpSwitch [18], which had been modified by replacing the *O. polymorpha* MAL promoter (OpMAL) with the *O. thermomethanolica* AOX promoter (OtAOX), using EcoRI (Thermo Scientific, Waltham, MA, USA) and SacI (Thermo Scientific, Waltham, MA, USA) to generate in-frame translation with the preceding alpha factor and the following 6X His tag. The resulting plasmids were checked by Sanger sequencing.

### 2.2. Yeast Transformation

Competent *O. polymorpha* TBRC 4839 (OP39) (Thailand Bioresource Research Center, Pathum Thani, Thailand) was prepared for transformation as described previously [20]. One µg NotI-linearized plasmids were transformed into the competent yeast cells by electroporation using the Bio-Rad gene Pulser system with a 2 mm cuvette with an electric field pulse: 1.5 kV/cm, 25 µF, 400 Ω. The transformants were selected on YPD agar supplemented with 100–1500 µg/mL of hygromycin and verified for gene integration by colony PCR with primers specific to the promoter and gene of interest regions of each vector.

### 2.3. Small-Scale Trial Expression and Flask-Scale Protein Production

For first-round screening, the cultures were grown from each clone (master plate) in 10 mL of YPD in a 50 mL conical tube at 37 °C with shaking at 250 rpm for 24 h. Cells were harvested and resuspended into 1 mL of BMMY medium [100 mM phosphate buffer pH 6.0, 1% *w*/*v* yeast extract, 2% *w*/*v* peptone, 1.34% YNB, 2 µg/L biotin, 1% *v*/*v* methanol] to induce protein expression at 26 °C with shaking at 250 rpm. Methanol was added to achieve a final concentration of 1% every 24 h. The full list of material sources is provided in Supplementary Table S2.

For shake-flask experiments, the cultures were grown from frozen glycerol stock in 5 mL YPD at 37 °C with shaking at 250 rpm overnight. Then, cultures were inoculated in 20 mL of BMGY [100 mM phosphate buffer pH 6.0, 1% *w*/*v* yeast extract, 2% *w*/*v* peptone, 1% *v*/*v* glycerol, 1.34% YNB, 2 µg/L biotin] at OD600 = 1 and cultured in a 250 mL flask at 37 °C with shaking at 250 rpm for 24 h. Cells were harvested and resuspended into 5 mL of BMMY medium with indicated compositions to induce protein expression at 26 °C with shaking at 250 rpm. During the subsequent 24–72 h, methanol was added to achieve a final concentration of 1% every 24 h. During optimization, parameters such as temperatures, pH levels or buffering agents were varied as indicated in each experiment.

### 2.4. Production of r-poIFN-α1 in a 5 L Fermenter

The recombinant *O. polymorpha* strain was maintained as glycerol stocks (20% *v*/*v*) at −80 °C. For seed culture preparation, 1 mL of frozen stock was inoculated into 20 mL of YPD medium and cultivated at 37 °C and 200 rpm for 18 h. A 10 mL aliquot of this culture was then transferred into 200 mL of YPD medium containing 20 g/L glucose as the carbon source and incubated at 37 °C with shaking at 250 rpm for 18 h. The resulting culture served as the inoculum for bioreactor fermentation.

Recombinant poIFN-α1 production was carried out in a 5 L laboratory-scale fermenter (BIOSTAT B Plus, Sartorius, Göttingen, Germany) using a methanol-inducible fed-batch process consisting of two phases: (i) a glycerol batch phase and (ii) methanol induction/production phase. During the batch phase, cells were cultivated in BMGY medium, prepared as previously described and supplemented with 40 g/L glycerol. Upon glycerol depletion, the culture was shifted directly to the induction/production phase. Methanol was first introduced as a bolus addition (~20 mL or 1% *v*/*v*) at the onset of induction at 17 h, followed by a stepwise-increasing continuous feed (approximately 50, 75, and 100 µL/min at successive intervals) to avoid methanol toxicity and sustaining the production of r-poIFN-α1. Aeration was maintained at 1 vvm throughout the batch and the induction/production phase, and dissolved oxygen was kept above 40% saturation by adjusting the agitation speed (500–1000 rpm) and supplementing with pure oxygen as needed. Fermentation temperature was controlled at 37 °C during the glycerol batch phase; upon methanol induction for IFN production, the culture temperature was reduced to 26 °C.

### 2.5. Recombinant poIFN Recovery

Prior to characterization, the culture supernatant containing r-poIFN underwent one-step ammonium sulfate precipitation. First, the supernatant was filtered through a 0.22-μm filter. Then, it was placed in a beaker, with constant stirring, placed in an ice bath. Ammonium sulfate was carefully and gradually added until 70% *w*/*v* salt was reached. The mixture was then centrifuged at 15,000× *g* at 4 °C for 10 min to collect the precipitated protein. The precipitated protein was then solubilized in PBS and dialyzed in PBS (100X volumes of the dissolved pellets) at 4 °C for at least 3 h. The resulting protein was concentrated to 10–15 mg/mL with the Amicon concentrator (Merck KGaA, Darmstadt, Germany, 10 kDa MWCO). Total protein concentration determination was performed with Bradford assay similar to the method published in USP <507> Protein determination procedures.

### 2.6. SDS-PAGE and Western Blotting

Samples were mixed with SDS-loading dye and boiled for 5 min before being separated on 12.5% sodium dodecyl sulfate-polyacrylamide gel (SDS-PAGE) prior to visualization with Coomassie Blue staining or Western blotting. For Western blot analysis, proteins were transferred to a nitrocellulose membrane using Mini Trans-Blot Cell (Bio Rad, Hercules, CA, USA). The membrane was probed with a mouse monoclonal IgG anti-His antibody (R&D systems, Minneapolis, MN, USA, cat. no. MAB050).

### 2.7. N-Linked Glycosylation Analysis

Twenty µL of 10 mg/mL r-poIFN proteins were treated with 4 µL PNGase F (New England Biolabs, Ipswich, MA, USA) or with sterilized water as a control (mock-treated) at 37 °C overnight in the non-denaturing buffer (1X GlycoBuffer (New England Biolabs) without NP-40 and no boiling). After treatment, treated and mock-treated recombinant porcine interferons were visualized with SDS-PAGE and Coomassie staining.

### 2.8. IFN Activity Assay

Unitage of r-poIFN was assigned using huIFN-α2a (NIBSC code: 95/650) as the in-plate reference standard in a laboratory-validated MDBK/VSV method modified from Rubinstein et al. 1981 [21]. Madin-Darby Bovine Kidney (MDBK) cell line (CCL-22) was procured from ATCC. Vesicular stomatitis virus (VSV) was produced by reverse genetics from the VSV-XN2 vector, a genomic plasmid of VSV Indiana serotype (a kind gift from Dr. Richard Vile, Mayo Clinic) [22]. IFN serial dilutions (200 μL each) were first deposited into 96-well plates, followed by 100 μL of 5.5 × 10^5^ cells/mL MDBK cells. The mixture was then incubated at 37 °C, 5% CO_2_ for 3 h. VSV (50 μL of 1.2 × 10^5^ TCID_50_/mL) was then added and the plate was incubated at 37 °C, 5% CO_2_ for further 20 h. Then, supernatant was removed, and an NBB (Naphthol Blue Black, cat. no. 195243, Sigma-Aldrich, St. Louis, MO, USA) solution was added. Uninfected cells were dyed with NBB at 37 °C, 5% CO_2_ for 1 h. Then, the NBB solution was removed, and the plate was washed with PBS twice. The leftover dye in each well was resolubilized with 100 μL of 50 mM NaOH on a shaker for 15 min at room temperature. OD620 was measured on a plate reader (BioTek microplate reader, Agilent, Santa Clara, CA, USA) and plotted as the sigmoidal curve and fitted to the four parameters logistic (4 PL) model to yield EC50 values, which could be converted to IU/mL with the in-plate reference standard. The method was validated for specificity, accuracy, precision following the ICH Q2(R2) guideline.

### 2.9. Antiviral Activity Measurement

VSV (Vesicular Stomatitis Virus): MDBK or PK-15 cells were maintained in DMEM High Glucose + 10% fetal bovine serum (FBS) (Cytiva Hyclone, Logan, UT, USA), in an incubator at 37 °C and 5% CO_2_. Recombinant VSV-mCherry was described previously [22]. For preliminary qualitative demonstration of bioactivities for recombinant porcine IFNs, cells were plated at 7.5 × 10^5^ cells/well in a 6-well plate and treated with 20 μg (MDBK) or 0.4–40 μg (PK-15) of r-poIFN for 24 h. The “untreated” controls were treated with PBS. Then, VSV-mCherry at multiplicity of infection (MOI) of 1 was incubated with cells for 1 h at 37 °C, 5% CO_2_. The inocula were then removed and cells were washed with PBS and supplied with fresh DMEM High Glucose + 10% FBS. At 24 h post-infection (hpi), cells were imaged on a fluorescence microscope (Olympus Corporation, Tokyo, Japan). The “mock” controls were cells plated on the same day but were not treated with r-poIFNs nor infected with the virus.

ASFV (African Swine Fever Virus): MA-104 cells were maintained in DMEM High Glucose + 10% FBS in an incubator at 37 °C and 5% CO_2_. ASFV strain AVCT-209 (cell-adapted field isolate in Thailand) was propagated in MA-104 cells. For anti-ASFV assay, MA-104 cells were plated at the concentration of 7.5 × 10^5^ cells/well in a 6-well plate and treated with 1 × 10^4^, 1 × 10^5^, 1 × 10^6^ IU/mL of r-poIFN-α1 for 24 h, while the “untreated” control was treated with PBS. The “Mock” control was not treated with IFN nor infected with the virus. Post-IFN treatment, the media was removed, cells were washed with PBS, and fresh DMEM High Glucose was added. ASFV strain AVCT-209 (MOI 0.1) was adsorbed onto the cells for 3 h at 37 °C and 5% CO_2_. Then, the inocula was removed and fresh DMEM High Glucose was added. Cytopathic effect (CPE) was monitored and imaged on a phase-contrast microscope every 24 hpi. Supernatants were collected at indicated times and quantified for ASFV titers by the qPCR method [23]. Four biological replicates were performed for statistical analysis.

PEDV (Porcine Epidemic Diarrhea Virus): Vero cells were maintained in OptiMEM + 10% FBS, in an incubator at 37 °C and 5% CO_2_. PEDV strain AVCT12 was propagated in Vero cells. For anti-PEDV assay, Vero cells were plated at the concentration of 7.5 × 10^5^ cells/well in a 6-well plate and treated with 1 × 10^4^, 1 × 10^5^, 1 × 10^6^ IU/mL of r-poIFN-α1 for 24 h, while the “untreated” control was treated with PBS. The “Mock” control was not treated with IFN nor infected with the virus. Then, treated or untreated cells were adsorbed with PEDV-AVCT12 (MOI 0.0001) for 1 h at 37 °C and 5% CO_2_. Then, the inocula was removed and the cells were washed twice with PBS. Fresh DMEM containing 1% TrypLE (protease aiding PEDV replication/infection; Thermo Scientific) was added. CPE was monitored and imaged on a microscope at indicated times. Supernatants were collected at indicated times and quantified for PEDV titers by the TCID_50_ method [24]. Four biological replicates were performed for statistical analysis.

PRRSV (Porcine Reproductive and respiratory Syndrome Virus): MARC-145 cells were maintained in OptiMEM + 10% FBS, in an incubator at 37 °C and 5% CO_2_. PRRSV strain 16CB02 was propagated in MARC-145 cells. For anti-PRRSV assay, MARC-145 cells were plated at the concentration of 6 × 10^5^ cells/well in a 6-well plate overnight. On the next day, cells were treated with 1 × 10^4^, 1 × 10^5^, 1 × 10^6^ IU/mL of r-poIFN-α1 for 24 h, while the “untreated” control was treated with PBS. The “Mock” control was not treated with IFN nor infected with the virus. Then, treated or untreated cells were adsorbed with PRRSV strain 16CB02 (MOI 0.01) for 1 h at 37 °C and 5% CO_2_. Then, the inocula was removed and fresh OptiMEM containing 10% FBS was added. CPE was monitored and imaged on a microscope at 48 and 72 hpi. Supernatants were collected at indicated times and quantified for PRRSV titers by the TCID_50_ method [24]. Two biological replicates were performed for preliminary analysis.

### 2.10. Statistical Analysis

Quantitative data for anti-ASFV and anti-PEDV activities were from four biological replicates. Each replicate was displayed as individual data points. Averaged log_10_ (titers), along with standard deviations, were plotted, and statistical significance was analyzed by paired t-test on log_10_-transformed titer values with GraphPad Prism version 10, GraphPad Software (San Diego, CA, USA). For anti-PRRSV activity, two biological replicates were performed to demonstrate preliminary quantitative data.

## 3. Results

### 3.1. Construction of Recombinant Yeast Hosts and Expression of Recombinant Porcine Interferons

Three porcine interferon candidates, each representing different types of interferons, were selected for trial expression in the *Ogataea polymorpha* TBRC 4839 (OP39) host and the expression vector pOpSwitch-AOX. *O. polymorpha*, formerly known as *Hansenula polymorpha*, is a thermotolerant yeast with Generally Recognized As Safe (GRAS) status and has been widely used as a production platform for several commercial biopharmaceuticals [25]. In our preliminary work, we have observed that, for some recombinant proteins with mammalian origins, the AOX promoter, which drives methanol oxidase expression in a closely related *O. thermomethanolica* (OT) yeast, performed notably better than the promoter driving the same gene but derived from *O. polymorpha* (pMOX) in both the homologous *O. thermomethanolica* TBRC 656 and the heterologous *O. polymorpha* TBRC 4839 (OP39) hosts (Supplementary Figure S2).

Codon-optimized synthetic DNAs bearing mature poIFN-encoding sequences were cloned into the expression vector pOpSwitch-AOX in-frame with the signal sequence and the 6X His tag. This vector comprises a MeOH-inducible promoter pAOX and a signal sequence of alpha mating factor from *O. thermomethanolica*. Each plasmid was linearized and transformed into OP39. About 30–50 transformants were selected for small-scale trial expression (1 mL BMMY in 50 mL conical tubes at standard conditions) for each poIFN type. Examples of recombinant porcine interferon expression were shown by Western blotting with anti-His tag antibody along with quantification of total proteins by Bradford assay (Figure 1). Several clones showed noticeably high expression compared to their peers. Generally, poIFN-α1 and poIFN-λ3 seem to be expressed at higher levels and in a larger number of transformants than poIFN-γ (Figure 1A–C and Table 1). Transformants with high recombinant protein expression, signified by intense bands in the blots or high total protein concentrations as measured by Bradford assay, were selected as producer clones for further study, namely clone 40 for poIFN-α1, clone 31 for poIFN-γ, and clone 20 for poIFN-λ3.

To confirm expression in these clones, we grew the selected producer clones in larger-scale flasks and observed protein production at different timepoints. Trial expression was performed in 5 mL BMMY in 250 mL flasks at 26 °C with 1% MeOH induction every 24 h. Recombinant protein production and secretion from each clone was monitored every 24 h with Western blotting. For poIFN-α1 and poIFN-λ3, gradual accumulation of proteins in the media could be observed as expected, while poIFN-γ were detected in small amounts on Day 1, undetectable in Day 2 and reappeared in small amounts in Day 3, hinting at protein instability problems in culture supernatant (Figure 2).

We next examined biochemical and biological properties of these recombinant interferons. We noted that all of them appeared heavier than their predicted molecular weights in Western blots, hinting at post-translational modification. The analysis of the amino acid sequences showed that all interferons contained at least one possible N-linked glycosylation site (Supplementary Figure S3). We tested if glycosylation was present and caused the shift from the expected molecular weights by incubation with PNGaseF. In the SDS-PAGE gels, all the recombinant proteins, especially poIFN-λ3 and poIFN-γ, appeared as smears (Figure 3, white arrows). However, when treated with PNGaseF, most of the proteins collapsed into one band with lower molecular weights, suggesting that all three types of recombinant porcine interferons were N-linked glycosylated (Figure 3, black arrows).

Next, antiviral activities of recombinant interferons were demonstrated by inhibition of vesicular stomatitis virus (VSV) in a standard cell-based assay [21]. Madin-Darby Bovine Kidney (MDBK) cells were treated with each type of recombinant porcine interferons (20 μg total protein) for 24 h prior to infection with the recombinant VSV expressing fluorescent mCherry protein. Cells were imaged at 24 h post-infection. In the untreated condition, red spots signifying prolific infection and replication of VSV-mCherry could be observed. In contrast, almost no red spots were detected in cells pre-treated with all three types of recombinant porcine interferons (Figure 4A). To simulate a closer biological situation, PK-15 cells, a representative for porcine cells, were used instead of a bovine cell. PK-15 cells were treated with various amounts of recombinant poIFN produced from OP39 for 24 h prior to VSV-mCherry infection. Cells treated with r-poIFN-α1 showed almost complete inhibition of viral replication at all tested concentrations (Figure 4B, top panel). Cells treated with r-poIFN-γ demonstrated roughly 10-fold weaker anti-VSV activity, while r-poIFN-λ3 at the same amount showed very little inhibition on VSV replication (Figure 4B, bottom and middle panels). In summary, these results demonstrated that recombinant interferons produced from the OP39 system possess antiviral activity, albeit with varying degrees depending on the type of cells and the type of interferons.

### 3.2. Optimization of Recombinant poIFN-α1 Production in Shake Flasks

Among the three types, poIFN-α1 demonstrated the highest potential as biotherapeutic agents, both for its biological activity and its production yield. Therefore, it was selected for further bioprocess development. We optimized the conditions to maximize the secreted expression of r-poIFN-α1, focusing especially on the pH level and induction temperatures; these two parameters are known from our past work to have important impact on heterologous protein expression in the *Ogataea* systems [26]. The standard condition, with pH 6.0 media at 30 °C, resulted in detectable r-poIFN-α1 expression on the second day but the level decayed on the next day. On the other hand, adjusting the pH of the BMMY induction media and lowering the induction temperature to 26 °C led to earlier and more stable expression over time (Figure 5A).

Next, we optimized for the buffering agent appropriate for the pH range identified. After biomass production, the culture was harvested and split equally for resuspension in two recipes of BMMY, pH 6.0. Protein production and secretion was monitored in shake flasks for 72 h. At the same amounts of loading sample, protein production in a modified BMMY (Na-citrate) showed the improved stability of r-poIFN-α1 when prolonging the production phase as presented in Figure 5B. The results in this part suggested that alteration of medium formulation variables such as pH or type/concentration of buffering agents could enhance the expression and stability of r-poIFN-α1.

### 3.3. Production of Recombinant poIFN-α1 in a 5 L Fermenter

Batch cultivation of recombinant OP39 achieved rapid exponential growth during the glycerol batch phase, with a specific growth rate (μ_max_) of 0.210 h^−1^ and a corresponding doubling time of 3.3 h (R^2^ = 0.982). Biomass increased from an initial 2.45 g/L to 18.2 g/L over 10 h, consuming glycerol from 47.96 g/L to 3.96 g/L, giving a biomass yield on glycerol (Y_sx_) of 0.358 g biomass g^−1^ glycerol.

Following the shift to methanol induction at 17 h, biomass continued to accumulate to a final concentration of 39.85 g/L over 54.5 h of induction (Figure 6A). Recombinant protein titer rose progressively throughout the induction phase to a final total protein concentration of 0.38 mg/mL (Figure 6B and Table 2), corresponding to a volumetric productivity (Q_p_) of 6.95 mg L^−1^ h^−1^ and a specific productivity (q_p_) of 0.21 mg g^−1^ h^−1^. The protein yield on biomass (Y_xp_) reached 27.7 mg protein g^−1^ biomass, and a total of 239.25 g of methanol was fed over the induction period. A biological replicate of this batch process also showed a broadly similar fermentation profile (Supplementary Figure S4). Across the two replicates, growth rate averaged 0.194 ± 0.021 h^−1^, final total protein concentration in culture supernatants averaged 0.45 ± 0.10 mg/mL, and volumetric productivity averaged 8.0 ± 1.5 mg L^−1^ h^−1^ (mean ± SD, *n* = 2), indicating a preliminary process reproducibility with moderate run-to-run variability. Full parameters for both replicates are provided in Supplementary Table S3.

### 3.4. Ogataea-Produced r-poIFN-α1 Inhibits Several Important Porcine Viruses

To demonstrate that *Ogataea*-produced r-poIFN-α1 exerts broad-spectrum antiviral activities, several important porcine viruses were selected to test their sensitivities to r-poIFN-α1 in cell culture assays as proof-of-concept experiments. First, we tested the antiviral activity against African swine fever virus (ASFV). MA-104 cells (African green monkey kidney epithelial cells) were pre-treated with varying doses of r-poIFN-α1 for 24 h prior to infection with ASFV (strain AVCT-209) and observed cytopathic effect (CPE) for 72 h. Compared to cells that were not treated with r-poIFN-α1 (untreated), significant reduction in CPE was observed in treated cells. ASFV titers as quantified by qPCR of the culture supernatants were decreasing in a dose-dependent manner (Figure 7A).

We next tested r-poIFN-α1 with porcine epidemic diarrhea virus (PEDV) in Vero cells. Cells were pre-treated with varying doses of r-poIFN-α1 for 24 h prior to infection with PEDV (strain AVCT12) and observed for syncytia as a proxy for PEDV replication. Compared to cells that were not treated with r-poIFN-α1 (untreated), fewer syncytia developed more slowly in cells treated with r-poIFN-α1 in a dose-dependent manner (Figure 7B, black arrows). r-poIFN-α1 treatment was effective at suppressing PEDV replication as 2–3-log decreases in titers were observed (Figure 7B).

Additionally, we performed preliminary tests for the anti-PRRSV activity using MARC-145 cells (monkey kidney epithelial cell). Cells were pre-treated with varying doses of r-poIFN-α1 for 24 h prior to infection with PRRSV (strain 16CB02; MOI 0.01) and observed for CPE for 48–72 h. Compared to cells that were not treated with r-poIFN-α1 (untreated), less CPE and reduced titers were observed in cells treated with r-poIFN-α1 (Figure 7C).

## 4. Discussion

Preparation for emerging or re-emerging swine viral outbreaks requires rapidly deployable tools for livestock management, such as broad-acting immunomodulators. This study evaluated the *Ogataea polymorpha* TBRC 4839 (OP39) yeast platform for producing recombinant porcine interferons (r-poIFNs) to assess its viability for biotherapeutic development. Generally, the primary metrics for evaluating protein expression hosts include overall royalties (freedom-to-operate), paths to regulatory approval, manufacturing cost and time, and control of post-translational modifications [27]. As demonstrated in this study, the OP39 system presents distinct advantages across these criteria, still with some rooms for improvement.

### 4.1. OP39 Host and Promoter Characteristics

OP39 was characterized and developed as a thermotolerant alternative to commercial *O. polymorpha* strains [18,19], offering full freedom-to-operate and eliminating the burden of costly licensing royalties for local or regional manufacturers. Furthermore, *O. polymorpha* (formerly *Hansenula polymorpha*) has a robust industrial safety record, including its use in licensed human biopharmaceuticals like the hepatitis B vaccines Recombivax HB and Heptavax^®^. This established safety profile provides a strong foundation that could ease the pathway to veterinary regulatory approval.

We demonstrated that all three r-poIFN types could be expressed using a heterologous methanol-inducible expression cassette (OtAOX) from the related *O. thermomethanolica*. Through extensive screening among constitutive (GAP) and inducible (OpMAL, OpMOX, OpFMD) promoters, we found that the constitutive and the sugar-inducible promoter could not produce detectable recombinant porcine interferons. Among different MeOH-inducible promoters, the heterologous expression cassette (OtAOX promoter) gave rise to the highest amount of recombinant protein production, even higher than the endogenous OpMOX promoter (Supplementary Figure S2). Past studies have shown that orthologous promoters derived from other yeasts could induce high levels of protein expression in *P. pastoris* [28,29,30]. Vice versa, promoters from *P. pastoris* could be functionally transferred to *H. polymorpha* host [31,32]. These results supported the functional compatibility among these yeast systems. Moreover, recent research has focused on the use of synthetic or engineered promoters to improve or precisely control expression of heterologous protein in several yeast systems, suggesting possible superiority over endogenous promoters [33,34].

### 4.2. r-poIFN-α1 Yield Comparison with Other Platforms

Among the three recombinant porcine interferons, type I r-poIFN-α1 demonstrated the best yield-to-activity profile and was selected for scale-up. In large-scale shake flasks and 5 L bioreactors, total protein yields in culture supernatant reached 0.3–0.5 g/L. Simple, one-step salt precipitation yielded total protein concentrations of 10–15 mg/mL with high specific biological activity (10^6^–10^7^ IU/mg). From a manufacturing cost and time perspective, this secretory yeast platform can be competitive. It requires a significantly simpler and faster downstream purification process compared to refolding proteins from bacterial inclusion bodies, and it is vastly less expensive than mammalian CHO cell platforms yielding similar crude titers ([35] and references therein).

While OP39 possesses several advantages as discussed above, its absolute yield for heterologous proteins remains modest compared to highly optimized *P. pastoris* platforms, which have reported poIFN-α1 yields of ~17 g/L [35], or fed-batch *H. polymorpha* with high-purity oxygen supply producing up to 5.8 g/L of human serum albumin [36]. This yield gap is likely driven by several contributing factors. First, we noted that the OP39 system might carry intrinsic limitations in producing more complex proteins. While industrial enzymes of bacterial origin (e.g., xylanase X11P) have achieved production yields of up to 6–7 g/L in this expression system [19], our prior use of the *Ogataea* expression system for a swine subunit vaccine (viral capsid protein) achieved only 0.12 g/L [26]. Second, the simple batch format itself limits both achievable biomass and the duration over which induction conditions can be tuned, relative to fed-batch or high-cell-density strategies. This strategy was partially forced due to the intrinsic instability of the target recombinant protein. In our efforts for fed-batch fermentation, we could not detect r-poIFN-α1 post-induction in high-cell-density conditions (K. Kocharin, unpublished results). Our bioreactor data highlighted this crucial process dynamic: while total protein concentration continued rising until 71.5 h, antiviral activity peaked earlier at 59.5 h before a slight decline. This discrepancy suggests that extended residence time under methanol induction—a condition previously associated with host protease activity and reduced protein stability in related methylotrophic yeast systems [26]—possibly led to partial proteolytic clipping or misfolding or secretion of non-target proteins. Consequently, at a later stage, biological activity is not strictly correlated with total protein concentration measurement. Therefore, harvest timing must be dictated by biological activity rather than maximum protein titer. To bridge the yield gap with commercial systems in the future, several strategies could be integrated into the OP39 platform, such as fed-batch high-cell-density fermentation, optimized gene dosage, engineered promoters and signal sequences, and CRISPR/Cas-aided targeted genome modifications to reduce host protease activity.

### 4.3. Glycosylation Profiles

A competitive advantage of the OP39 platform for r-poIFN-α1 could be its post-translational modification profile. Yeasts like *S. cerevisiae* and *P. pastoris* frequently add high-mannose N-glycans, which are structurally distinct from mammalian glycans. In *Pichia*, r-poIFN-α1 exhibits significant hyperglycosylation, with large molecular weight species (40–50 kDa) present in amounts equal to the lightly glycosylated fractions [35]. In contrast, our preliminary observations here demonstrated that OP39-derived r-poIFN-α1 showed minimal hyperglycosylation, consistently resolving as doublets around 20 kDa. Both works demonstrated that glycosylation did not affect biological activities measured in cell-based assays ([35] and Supplementary Figure S5); however, in clinical applications, hyperglycosylation can trigger rapid clearance, unwanted immunogenicity, and inflammatory responses via mannose-binding lectin pathways [37]. Therefore, avoiding hyperglycosylation is a critical advantage that reduces the risk of adverse immune reactions and eases the analytical burden of ensuring therapeutic consistency during biomanufacturing. Further detailed glycan analysis of the recombinant products from the OP39 system and in vivo experiments to generate full safety profiles will be required to confirm these attributes.

### 4.4. Biological Activity and Cell-Type Specificity of r-poIFNs

Beyond manufacturing metrics, biological activity is a critical factor that dictates decisions for developing clinical biotherapeutics. Our cell-based assays confirmed the distinct biological profiles of the expressed IFNs. In our VSV infection assays, standard MDBK cells responded robustly to all r-poIFNs, whereas PK-15 cells exhibited differential responses depending on the IFN type. This divergence is possibly driven by receptor distribution. Type I IFNs exert broad antiviral activity across most cell types, whereas Type III IFNs require the IFNLR1 receptor, whose functional in vitro expression varies significantly among epithelial cell lines. Consequently, reported poIFN-λ3 activity in PK-15 cells is mixed: while it partially protected against an attenuated pseudorabies virus (PRV) lacking IFN antagonists [38], it failed to suppress wildtype PRV—a scenario where exogenous r-poIFN-α succeeded [39]. This lack of type III efficacy against wildtype virus in PK-15 cells closely parallels our own observations with VSV. In contrast, poIFN-λ3 efficiently limits PRV in respiratory and intestinal cells where IFNLR1 is abundantly expressed [39]. Therefore, while type I IFNs offer broad-spectrum utility, type III IFNs may be better suited for specialized mucosal applications. Ultimately, in vivo trials remain essential to validate the safety and therapeutic efficacy of these OP39-produced r-poIFNs.

### 4.5. Limitations and Future Directions

The major limitations of this study are (i) the relatively low yield of r-poIFN-α1, despite intensive producer clone selection and pre-pilot bioprocess optimization, (ii) detailed glycan analysis on the recombinant product, and (iii) the lack of pre-clinical tests in pigs to support safety and efficacy. Despite current yield shortfalls relative to heavily engineered commercial yeast strains, the OP39 platform offers a viable alternative for recombinant veterinary biotherapeutics. Based on the results in this study, it shows potential to satisfy the core criteria for expression host selection: low manufacturing cost with simple downstream processing, relatively low hyperglycosylation, a strong safety record, and full freedom-to-operate. Future efforts will focus on bioprocess optimization to maximize functional yield and in vivo efficacy trials to validate these promising swine health management tools.

## 5. Conclusions

This study offers *Ogataea polymorpha* OP39 as a promising proof-of-concept production platform with full freedom-to-operate for the regional production of recombinant veterinary biotherapeutics. We demonstrated the expression and secretion of three major types of recombinant porcine interferons (poIFN-α1, poIFN-γ, and poIFN-λ3) using a methanol-inducible expression system. Among the evaluated candidates, the type I interferon poIFN-α1 demonstrated the most promising yield-to-activity profile and was successfully scaled up in a 5 L bioreactor. Crucially, the OP39-produced r-poIFN-α1 exhibited relatively low hyperglycosylation often associated with other yeast systems and displayed antiviral activity in vitro against critical swine pathogens, including ASFV, PEDV, and PRRSV. While the current absolute yields are modest compared to heavily engineered commercial strains, the platform’s distinct advantages—namely its full freedom-to-operate, simple downstream processing, and established safety record—make it a potential candidate for biomanufacturing. Future efforts will focus on advanced bioprocess optimization, such as high-cell-density fed-batch fermentation, to close the yield gap, as well as in vivo efficacy trials to fully validate these interferons as practical tools for swine health management.

## Figures and Tables

**Figure 1:**
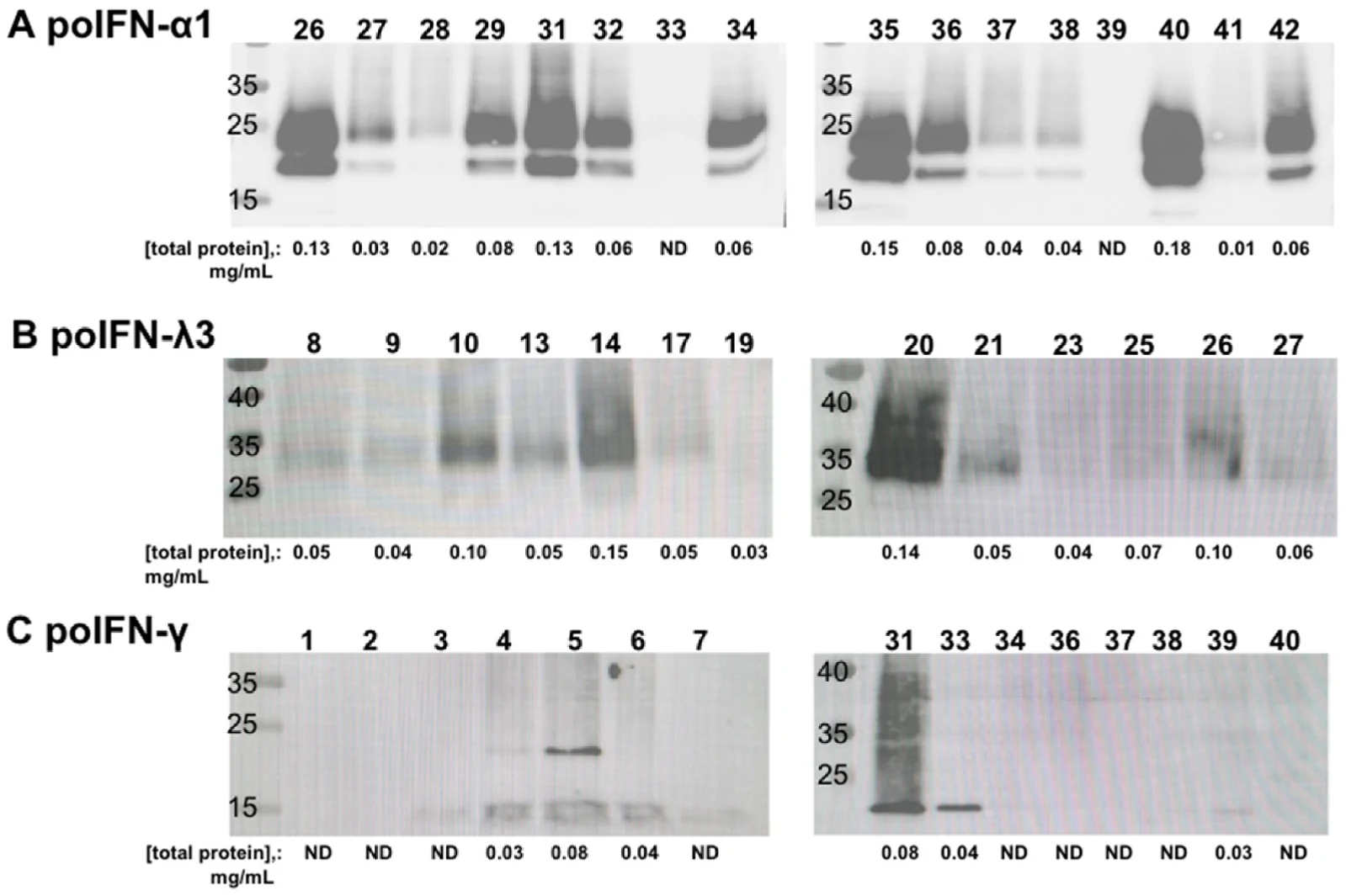
Selection of producer clones for poIFN-α1 (**A**), poIFN-λ3 (**B**) and poIFN-γ (**C**). Transformants were grown in YPD and induced for protein expression in BMMY. Equal amounts of culture supernatants were loaded on to SDS-PAGE gels, and protein expression was visualized by Western blotting with anti-His tag antibody. Numbers on top of gels signified the transformants’ numbers. Total protein concentrations measured from culture supernatant with the Bradford method were denoted at the bottom of each lane. Screening was performed in one biological replicate, and images were selected representatives from about 30–50 clones each. ND, not detected.

**Figure 2:**
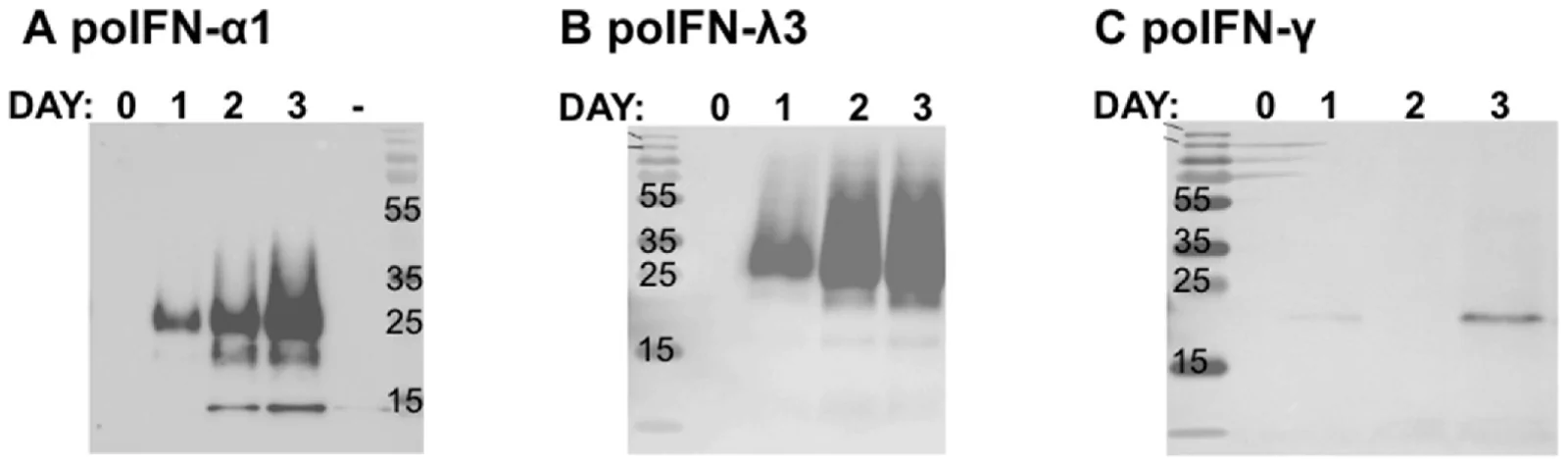
Trial expression for poIFN-α1 (**A**), poIFN-λ3 (**B**) and poIFN-γ (**C**). Producer clones for each protein were grown in BMGY and induced for protein expression in BMMY, with addition of 1% MeOH every 24 h. Supernatants from Days 0, 1, 2, 3 post-induction were monitored for protein expression with SDS-PAGE and Western blotting with anti-His tag antibody. Images were representative of two biological replicates.

**Figure 3:**
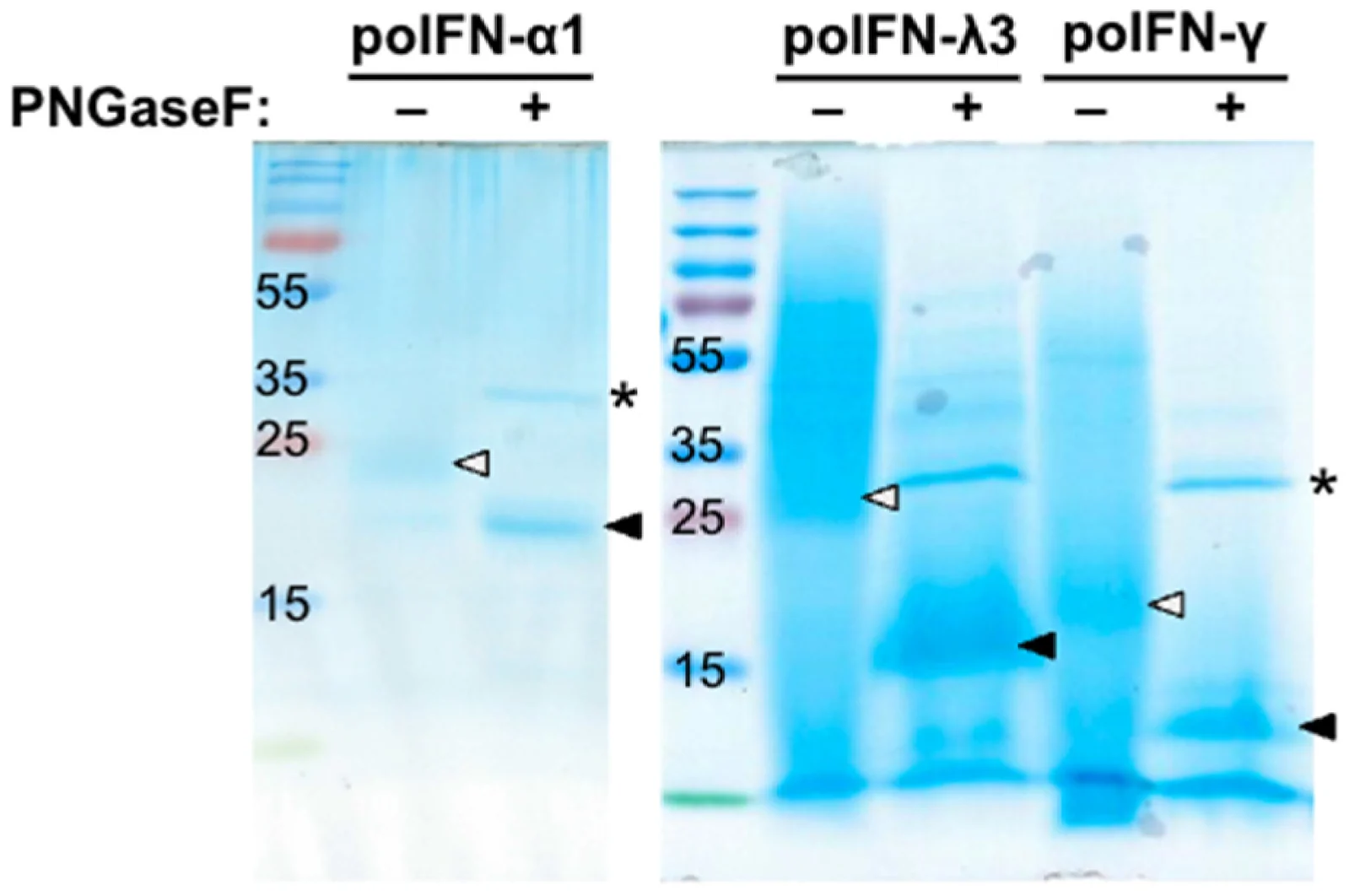
Glycosylation analysis by PNGase F treatment of recombinant porcine interferons produced by the OP39 system. White arrows denote the major bands of recombinant porcine interferons. Black arrow denotes the deglycosylated recombinant porcine interferons. Asterisk denotes PNGase F enzyme (MW 35.5 kDa). Images were representative of two biological replicates.

**Figure 4:**
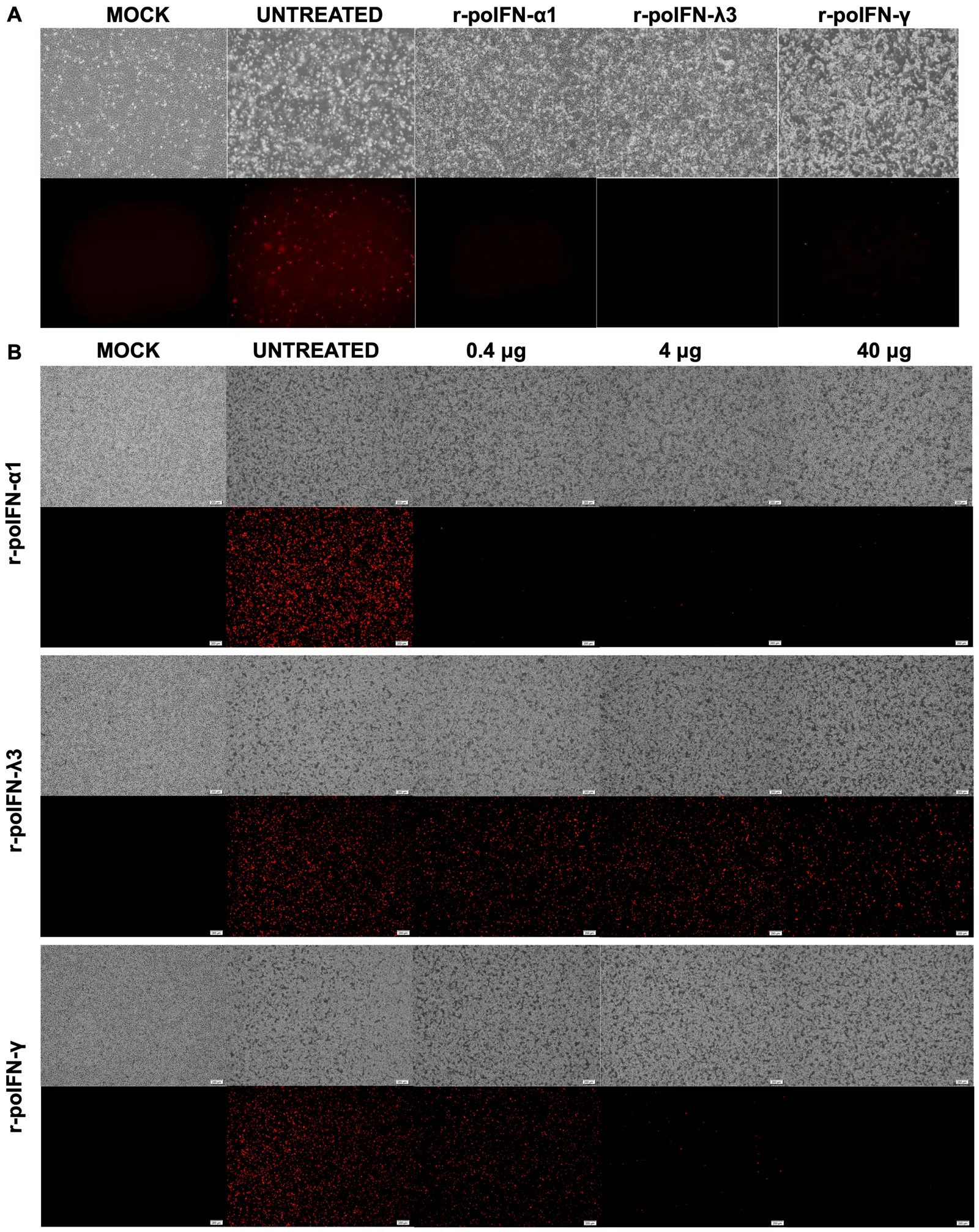
Preliminary antiviral (VSV) activity tests of *Ogataea*-produced recombinant porcine interferons in MDBK cells (**A**) or PK-15 cells (**B**). Cells were pre-treated with recombinant porcine interferons for 24 h prior to infection with recombinant VSV expressing mCherry fluorescent protein. Cells were imaged at 24 h post-infection by a fluorescence microscope (bright-field to show cell morphology and red fluorescence to show viral replication). Scale bar, 200 μm. Images were representative of two biological replicates.

**Figure 5:**
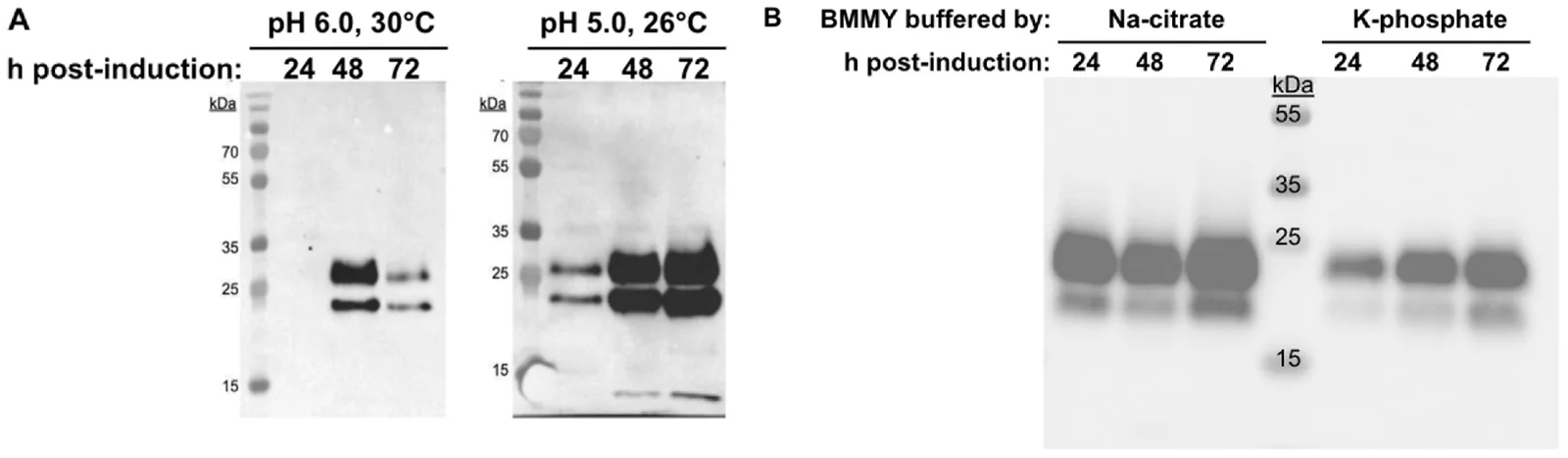
Optimization of r-poIFN-α1 expression conditions. (**A**) Comparison of r-poIFN-α1 expression levels between standard (pH 6.0, 30 °C) and optimized (pH 5.0, 26 °C) conditions. (**B**) Modified Na-citrate buffered medium (left) can improve r-poIFN-α1 stability in culture supernatant, compared to standard BMMY with K-phosphate, pH 5.0 (right). Culture supernatants from small-scale shake flasks were analyzed by Western blotting with anti-His tag antibody. Samples were taken at indicated timepoint (h post-induction).

**Figure 6:**
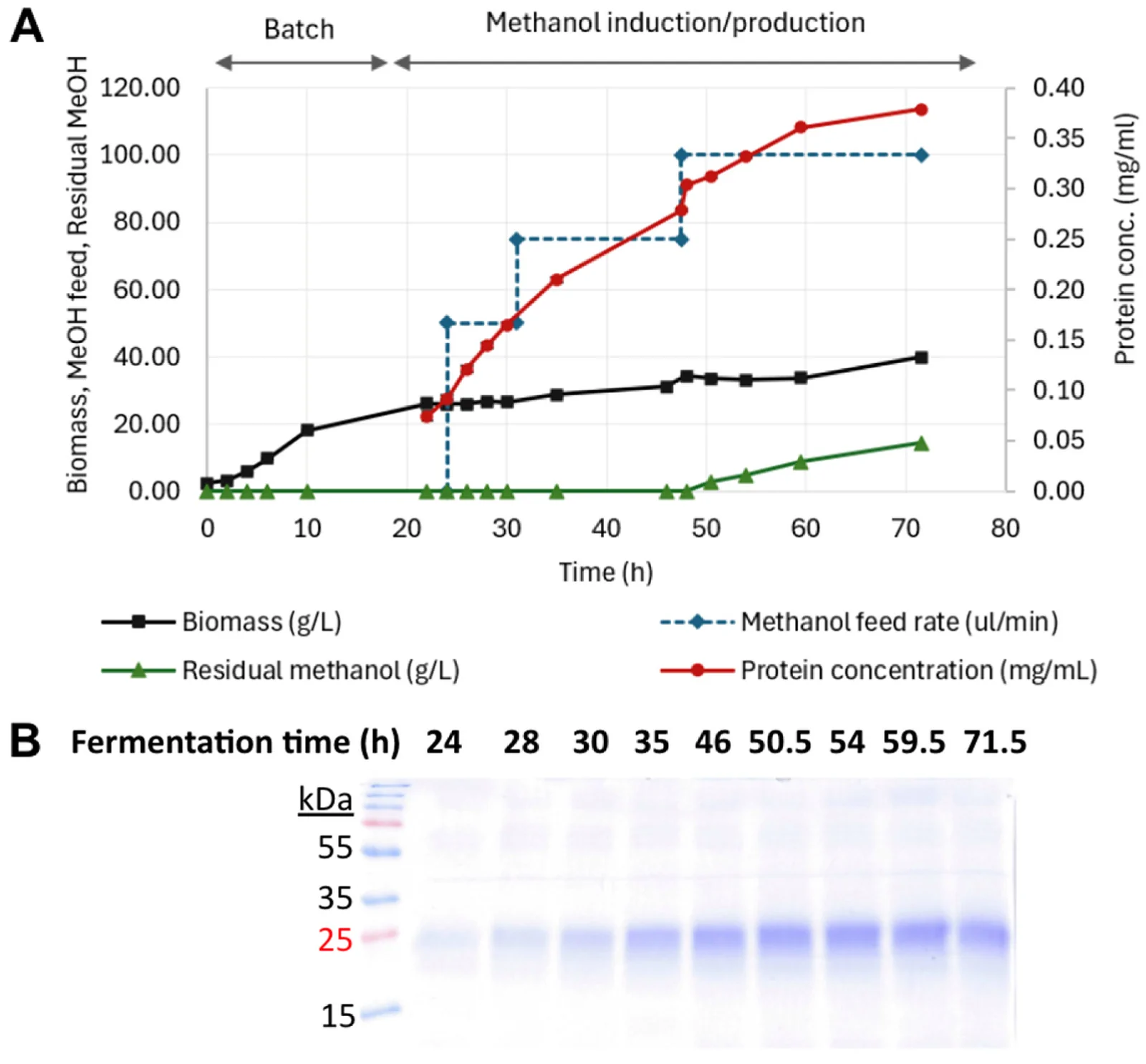
Batch fermentation and expression profile of r-poIFN-α1 produced by *O. polymorpha*. (**A**) Time course of biomass (black squares, g/L), methanol feed rate (blue dashed diamonds, µL/min), residual methanol (green triangles, g/L), and total protein concentration in culture supernatant (red circles, mg/mL) throughout the glycerol batch phase (0–~17 h) and subsequent methanol induction/production phase (~17–71.5 h), as indicated by the phase brackets above the plot. (**B**) SDS-PAGE (Coomassie-stained) analysis of culture supernatant samples collected at the indicated fermentation times.

**Figure 7:**
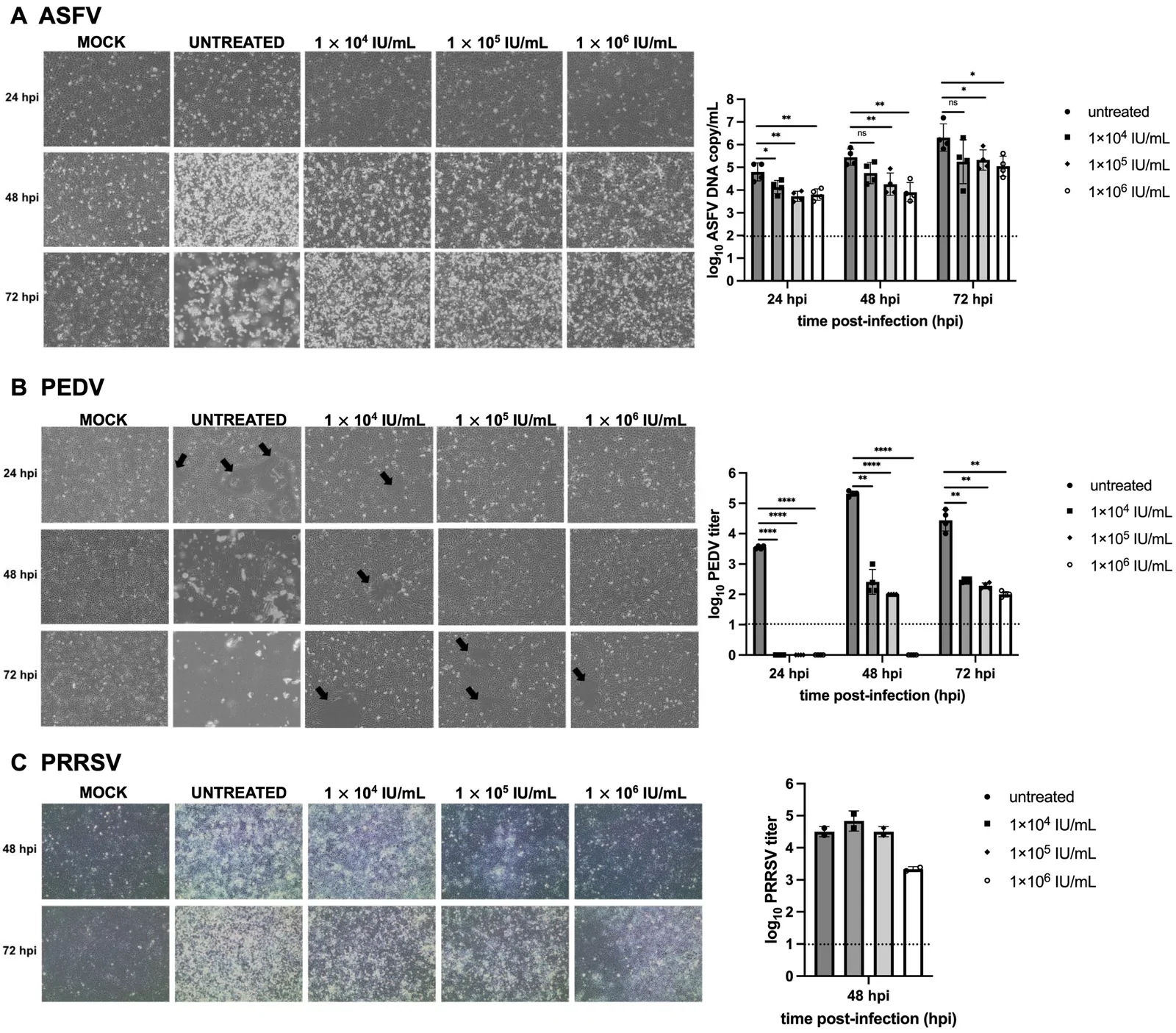
Antiviral activities of *Ogataea*-produced r-poIFN-α1. (**A**) Anti-ASFV activity of r-poIFN-α1 on MA-104 cells as observed by reduction in cytopathic effect and ASFV titers quantified by qPCR. (**B**) Anti-PEDV activity of r-poIFN-α1 on Vero cells as observed by reduction in syncytia (black arrows) and PEDV titers quantified by TCID_50_. (**C**) Anti-PRRSV activity of r-poIFN-α1 on MARC-145 cells as observed by reduction in cytopathic effect and PRRSV titers quantified by TCID_50_. Images were representative of four biological replicates for ASFV and PEDV and two biological replicates for PRRSV. Data points represent each biological replicate, while bar graphs and error bars represent average ± SD. Statistical significance was determined for (**A**,**B**) by paired t-test on log_10_-transformed titer values. ns, *p* > 0.05, * *p* < 0.05, ** *p* < 0.01, **** *p* < 0.0001. Dotted lines represent limits of detection.

**Table 1:** Summary of the trial expression results for recombinant porcine interferons in OP39.

Protein	Number of Tested Clones	Number of Clones with poIFN Expression Bands	Number of Clones with Total Protein ≥ 0.1 mg/mL
poIFN-α1	55	50 (91%)	11 (20%)
poIFN-λ3	28	18 (64%)	4 (14%)
poIFN-γ	27	6 (22%)	0 (0%)

**Table 2:** Production of r-poIFN-α1 in 5 L fermentation trial shown in Figure 6.

Fermentation Time (h)	Production Time (h)	Total Protein Concentration (mg/mL)	IFN Activity (×10^6^ IU/mL)
24	7	0.09	0.62
28	11	0.14	2.03
30	13	0.17	3.00
35	18	0.21	3.05
46	29	0.28	3.13
50.5	33.5	0.31	4.21
54	37	0.33	3.90
59.5	42.5	0.36	5.69
71.5	54.5	0.38	4.04

## Data Availability

The original contributions presented in this study are included in the article/Supplementary Materials. Further inquiries can be directed to the corresponding author.

## Supplementary Materials

The following supporting information can be downloaded at https://www.mdpi.com/article/10.3390/microorganisms14102150/s1, Figure S1: Codon-optimized coding sequences for mature porcine interferons; Figure S2: Comparison of r-poIFN-α1 expression in recombinant OP39 under the *O. polymorpha* MOX and *O. thermomethanolica* AOX promoters; Figure S3: Prediction of N-link glycosylation on poIFN-α1, poIFN-λ3 and poIFN-γ; Figure S4: A biological replicate of a batch fermentation and expression profile of r-poIFN-α1 produced by *Ogataea polymorpha*; Figure S5: Biological activity of deglycosylated r-poIFN-α1 is not significantly different from that of OP39-derived glycosylated r-poIFN-α1; Table S1: Amino acid sequences of mature porcine interferons in this study; Table S2: Full list of material sources; Table S3: Summary of growth and r-poIFN-α1 production parameters in fermentation.

## Abbreviations

The following abbreviations are used in this manuscript:

ASFV	African swine fever virus
CPE	Cytopathic effect
FMDV	Foot-and-Mouth Disease Virus
GRAS	Generally Recognized As Safe
hpi	Hours post-infection
IFN	Interferon
IU	International Unit
MDBK	Madin-Darby Bovine Kidney
OP39	*Ogataea polymorpha* TBRC 4839
OT	*Ogataea thermomethanolica*
PAM	Porcine pulmonary alveolar macrophage
PEDV	Porcine epidemic diarrhea virus
PRRSV	Porcine Reproductive and Respiratory Syndrome Virus
PRV	Pseudorabies virus
r-poIFN	Recombinant porcine interferon
SDS-PAGE	Sodium dodecyl sulfate-polyacrylamide gel
VSV	Vesicular stomatitis virus
