# Supplementary material for: Ogataea Yeast Platform for Recombinant Porcine Interferon-Alpha 1 Production: Toward Alternative Biotherapeutics for Swine Health Management
Source: Microorganisms. 2026 Sep 23;14(10):2150. doi: 10.3390/microorganisms14102150 (PMC13707865; doi:10.3390/microorganisms14102150)
Supplement: Supplementary file 1 [file microorganisms-14-02150-s001.zip › microorganisms-4516715-supplementary.pdf]

Supplementary Figure S1

Alpha1:

TGC GAT TTG CCC CAG ACT CAT TCT TTG GCC CAT ACT CGT GCT TTG AGG TTG TTA GCT
CAG ATG AGA AGG ATA TCT CCA TTT TCT TGT CTT GAC CAC AGA AGA GAT TTC GGT TCA
CCA CAT GAA GCT TTT GGT GGC AAT CAA GTT CAG AAA GCA CAA GCT ATG GCC TTG GTT
CAC GAA ATG TTA CAA CAA ACT TTC CAA CTT TTC TCC ACA GAA GGT TCC GCT GCT GCA
TGG AAT GAA TCA TTG CTT CAC CAG TTC TGT ACC GGA CTG GAC CAA CAG TTA AGA GAT
CTT GAG GCC TGC GTG ATG CAG GAA GCT GGA CTG GAG GGT ACA CCT TTG CTG GAG GAG
GAC TCT ATT TTG GCC GTC CGT AAA TAC TTT CAC AGA CTG ACT TTG TAC TTA CAG GAG
AAG TCC TAT TCT CCA TGC GCA TGG GAG ATC GTA AGA GCA GAG GTC ATG CGT AGT TTT
AGT TCT TCT AGG AAC TTG CAA GAC AGA TTG AGA AAG AAA GAA

Lambda3:

GAA GCA TTG CGT GCT TTA CCA GGT GCT AGG GGT TGT CAC CTT GCC CAA TTT AAA TCT
CTT TCT CCT CAG GCA TTA CAG GCC TTT AAG AGA GCT AAA GAC GCA TTC GAG GAG TCA
TTG TTG GAG GAC TGG AAC TGT TCT TCT CGT ATT TTC CCT CGT TCA AGA GAC TTG AAG
CAG TTG CAG GTG TGG GAA AGA CCA GTC GCT CTT GAA GCT GAA GTT GCC CTT ACT TTG
TCT GTT TTG GGT TCT TTG GCC AAT TCC TCA CTG CAC TCT TCC TTA GAT CAA CCA TTA
CAC ACC TTG AGA CAT ATA CAT GCT CAG TTA CAA GCC TGC GTC CCA GCT CAA CCA ATG
GCT GGT CCT AGA CCA AGA GGA AGA CTG CAT CAT TGG TTA CAC AGA TTG CAG GAA GCA
CAA AAA AAA GAG CCA CAA TCT TGT TTG GAG GCA TCT GTT ATG TTC AAT TTG TTT AGG
CTG TTG ACT AGG GAT TTG AAG TGC GTT GCT TCT GGA GAT CTG TGC GTG

Gamma:

TGC CAA GCT CCC TTC TTC AAG GAA ATA ACT ATC CTG AAA GAC TAC TTT AAC GCC TCA
ACA TCT GAT GTT CCA AAC GGT GGT CCA TTG TTC CTT GAG ATT TTG AAA AAT TGG AAA
GAG GAA TCC GAT AAG AAA ATC ATC CAA TCC CAG ATC GTT TCT TTT TAT TTT AAG TTT
TTT GAG ATC TTC AAG GAC AAT CAA GCA ATA CAA AGA TCT ATG GAC GTC ATT AAA CAG
GAT ATG TTC CAA AGA TTC CTT AAT GGT AGT TCT GGT AAG TTG AAT GAC TTC GAG AAA
TTG ATA AAG ATT CCA GTG GAC AAC TTA CAA ATT CAG AGA AAG GCC ATA AGT GAA TTG
ATA AAA GTG ATG AAT GAT CTG TCT CCT AGG TCT AAC TTA AGA AAA AGG AAG AGA TCA
CAG ACC ATG TTT CAG GGA CAG CGT GCT TCC AAG

Supplementary Figure S1 Codon-optimized coding sequences for mature porcine interferons.

Supplementary Table S1 Amino acid sequences of mature porcine interferons in this study.

Protein	Amino acid sequences
poIFN-α1	CDLPQTHSLAHTRALRLLAQMRRISPFSCLDHRRDFGSPHEAFGGNQVQKAQAMAL<br>VHEMLQQTFQLFSTEGSAAAWNESLLHQFCTGLDQQLRDLEACVMQEAGLEGTPLL<br>EEDSILAVRKYFHRLTLYLQEKSYSPCAWEIVRAEVMRSFSSSRNLQDRLRKKE
poIFN-λ3	EALRALPGARGCHLAQFKSLSPQALQAFKRAKDAFEESLLEDWNCSSRIFPRSRDL<br>KQLQVWERPVALEAEVALTLSVLGSLANSSLHSSLDQPLHTLRHIHAQLQACVPAQ<br>PMAGPRPRGRLHHWLHRLQEAQKKEPQSCLEASVMFNLFRLLTRDLKCVASGDLCV
poIFN-γ	CQAPFFKEITILKDYFNASTSDVPNGGPLFLEILKNWKEESDKKIIQSQIVSFYFK<br>FFEIFKDNQAIQRSMDVIKQDMFQRFLNGSSGKLNDFEKLIKIPVDNLQIQRKAIS<br>ELIKVMNDLSPRSNLRKRKRSQTMFQGQRASK

Supplementary Table S2 Full list of material sources

Material	Full name	Source
YPD	Yeast-Peptone-Dextrose	2% w/v Peptone,<br>1% w/v Yeast extract,<br>2% w/v Glucose
yeast extract	-	GIBCO, USA
peptone	-	GIBCO, USA
glycerol	-	Carlo Erba, Italy
YNB	Yeast Nitrogen base (without amino acids)	Difco, USA
biotin	-	Sigma-Aldrich, USA
MeOH	methanol	Carlo Erba, Italy
Ammonium sulfate	-	Sigma-Aldrich, USA
NP-40	Nonylphenoxypolyethoxylethanol	Thermo Scientific, USA
Glucose	-	Sigma-Aldrich, USA

Supplementary Figure S2

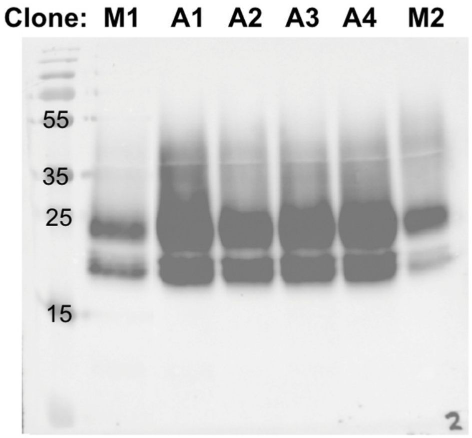

Supplementary Figure S2 Comparison of r-poIFN-α1 expression in recombinant OP39 under the *O. polymorpha* MOX and *O. thermomethanolica* AOX promoters. Different numbers at the top of the gel represent culture supernatants from different transformants cultured in the same condition. M1 and M2 were clones with MOX promoters; A1-A4 were clones with AOX promoters.

Supplementary Figure S3

A

```
Name: po-IFN-a1          Length:  166
CDLPQTHSLAHTRALRLLAQMRRISPFSCLDHRRDFGSPHEAFGGNQVQKAQAMALVHEMLQQTFQLFSTEGSAAAWNES      80
LLHQFCTGLDQQLRDLEACVMQEAGLEGTPLLEEDSILAVRKYFHRLTLYLQEKSYSPCAWEIVRAEVMRSFSSSRNLQD     160
RLRKKE                                                                              240
.............................................................................N..      80
................................................................................     160
......                                                                              240

(Threshold=0.5)
----------------------------------------------------------------------
SeqName      Position  Potential   Jury    N-Glyc
                                 agreement result
----------------------------------------------------------------------
Sequence       78 NESL   0.5780     (7/9)    +
----------------------------------------------------------------------
```

B

```
Name: po-IFN-L3          Length:  176
EALRALPGARGCHLAQFKSLSPQALQAFKRAKDAFEESLLEDWNCSSRIFPRSRDLKQLQVWERPVALEAEVALTLSVLG      80
SLANSSLHSSLDQPLHTLRHIHAQLQACVPAQPMAGPRPRGRLHHWLHRLQEAQKKEPQSCLEASVMFNLFRLLTRDLKC     160
VASGDLCVELHHHHHH                                                                    240
...........................................N....................................      80
...N............................................................................     160
................                                                                    240

(Threshold=0.5)
----------------------------------------------------------------------
SeqName      Position  Potential   Jury    N-Glyc
                                 agreement result
----------------------------------------------------------------------
Sequence       44 NCSS   0.6918     (9/9)    ++
Sequence       84 NSSL   0.5009     (5/9)    +
----------------------------------------------------------------------
```

C

```
Name: po-IFN-g           Length:  152
CQAPFFKEITILKDYFNASTSDVPNGGPLFLEILKNWKEESDKKIIQSQIVSFYFKFFEIFKDNQAIQRSMDVIKQDMFQ      80
RFLNGSSGKLNDFEKLIKIPVDNLQIQRKAISELIKVMNDLSPRSNLRKRKRSQTMFQGQRASKELHHHHHH             160
................N...............................................................      80
...N....................................................................             160

(Threshold=0.5)
----------------------------------------------------------------------
SeqName      Position  Potential   Jury    N-Glyc
                                 agreement result
----------------------------------------------------------------------
Sequence       17 NAST   0.5593     (6/9)    +
Sequence       84 NGSS   0.5914     (7/9)    +
----------------------------------------------------------------------
```

Supplementary Figure S3 Prediction of N-link glycosylation on poIFN-α1 (A), poIFN-λ3 (B) and poIFN-γ (C). Results are generated by NetNGlyc - 1.0. The predicted glycosylation motifs are in colors, with the asparagine residues in red. The numbers of jury agreement signify the likelihood of glycosylation at such motifs.

Supplementary Figure S4

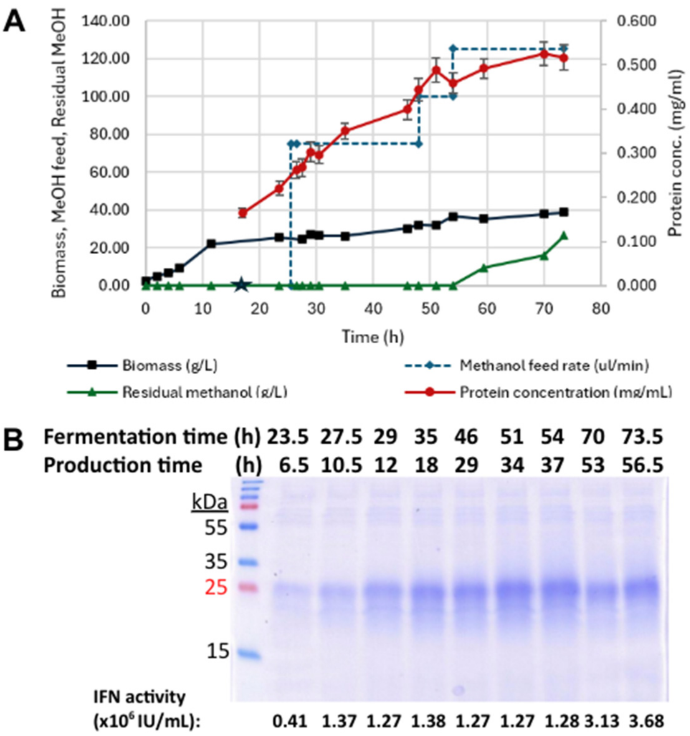

Supplementary Figure S4 A biological replicate of a batch fermentation and expression profile of r-poIFN-α1 produced by *Ogataea polymorpha*. (A) Biomass (black squares, g/L, left axis) increased steadily during the glycerol batch phase and continued to accumulate more slowly after methanol induction, reaching a maximum of ~40 g/L before plateauing. Methanol feed rate (blue diamonds, µL/min, left axis) was increased stepwise from induction onset to sustain protein expression while limiting methanol toxicity. Residual methanol (green triangles, g/L, left axis) remained near zero throughout most of the induction phase, rising only after ~48 h. Recombinant protein concentration (red circles, mg/mL, right axis) rose progressively from induction onset, reaching 0.52 mg/mL by 70 h. A biological replicate of batch process showed a broadly similar fermentation profile, with μ_{max} = 0.179 h^{-1} (t_d = 3.9 h), Y_{sx} = 0.70 g/g, and a final protein titer of 0.52 mg/mL (Q_P = 9.13 mg L^{-1} h^{-1}, q_P = 0.284 mg g^{-1} h^{-1}). (B) SDS-PAGE (Coomassie-stained) analysis of culture supernatant samples collected at the indicated fermentation and production (post-induction) times. Numbers below each lane indicated the IFN activity in International Unit per milliliter culture supernatant (IU/mL) as measured by the validated MDBK/VSV method.

Supplementary Table S3 Summary of growth and r-poIFN-α1 production parameters in fermentation.

Parameter	Rep1 (Figure6)	Rep2 (Supplementary)
**1. Growth-phase (glycerol batch) kinetics**		
Specific growth rate, μ_{max} (ln-linear regression of biomass vs. time) [h^{-1}]	0.2096	0.1793
Regression R^2	0.982	0.968
Biomass at end of batch phase [g/L]	18.20	21.95
Initial glycerol concentration, S_0 [g/L]	47.96	40.53
**2. Induction-phase (methanol) production kinetics**		
Induction start time (first methanol feed) [h]	17.0	17.0
Induction end time (last sampling point) [h]	71.5	73.5
Induction duration, Δt [h]	54.5	56.5
Biomass at induction start, X_0,ind [g/L]	26.15	25.45
Final biomass, X_f [g/L]	39.85	38.85
Max. protein titer, [mg/L]	379.0 ± 0.001	525.0± 27.0
Final protein titer, P_f (P_0,ind assumed ≈ 0) [mg/L]	379.0 ± 0.001	515.8± 28.6
Volumetric productivity, $Q_p = P_f / \Delta t$ [mg protein / L / h]	6.95	9.13
Average biomass during induction, X_{avg} [g/L]	33.00	32.15
Specific productivity, $q_p = Q_p / X_{avg}$ [mg protein / g biomass / h]	0.21	0.28
Protein yield on biomass, $Y_{p/x} = P_f / (X_f - X_0$,ind) [mg protein / g biomass]	27.66	38.50
Total methanol fed during induction (source data, cumulative) [g]	239.3	256.4
Specific methanol consumption rate, qMeOH = MeOH_total / (X_{avg} × Δt) [g MeOH / g biomass / h]	0.133	0.141
**3. Overall process summary**		
Total process time [h]	71.5	73.5
Maximum OD_{600} reached	185.6	215.0
Maximum biomass (DCW) reached [g/L]	39.85	38.85
Final total protein concentration (mg/mL units) [mg/mL]	0.38	0.52

Supplementary Figure S5

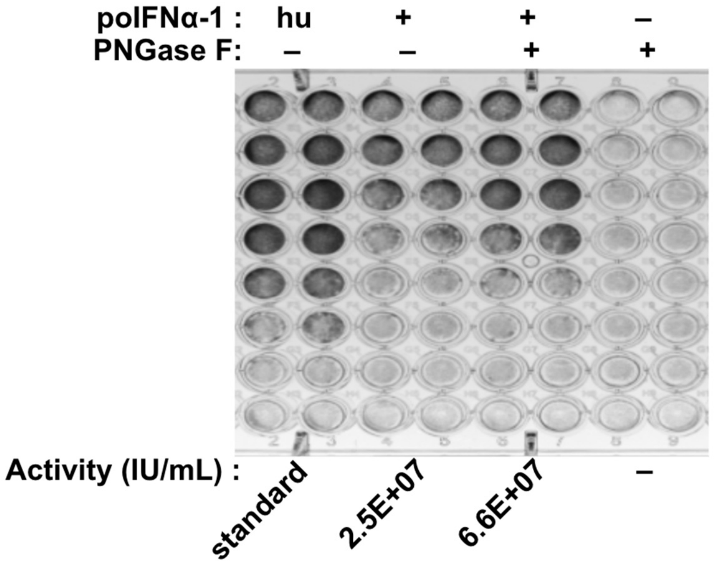

Supplementary Figure S5 Biological activity of deglycosylated r-poIFN-α1 is not significantly different from that of OP39-derived glycosylated r-poIFN-α1. The image depicted the MDBK/VSV method, where colored wells represented attached uninfected MDCK cells dyed with the NBB solution (protected by IFN) and uncolored wells represented detached MDCK cells that were infected and killed by VSV. 'hu' is WHO international standard for human r-IFN-α2a, which was used as an in-plate reference for unitage.
